# 3,5 Diiodo-l-Thyronine (T_2_) Promotes the Browning of White Adipose Tissue in High-Fat Diet-Induced Overweight Male Rats Housed at Thermoneutrality

**DOI:** 10.3390/cells8030256

**Published:** 2019-03-18

**Authors:** Rosalba Senese, Federica Cioffi, Rita De Matteis, Giuseppe Petito, Pieter de Lange, Elena Silvestri, Assunta Lombardi, Maria Moreno, Fernando Goglia, Antonia Lanni

**Affiliations:** 1 Department of Environmental, Biological and Pharmaceutical Sciences and Technologies, University of Campania “L. Vanvitelli”, 81100 Caserta, Italy; giuseppe.petito@unicampania.it (G.P.); pieter.delange@unicampania.it (P.d.L.); 2 Department of Sciences and Technologies, University of Sannio, 82100 Benevento, Italy; federica.cioffi@unisannio.it (F.C.); silvestri@unisannio.it (E.S.); moreno@unisannio.it (M.M.); goglia@unisannio.it (F.G.); 3 Department of Biomolecular Sciences, Urbino University, 61029 Urbino, Italy; rita.dematteis@uniurb.it; 4 Department of Biology, University of Naples Federico II, 80126 Naples, Italy; aslombar@unina.it

**Keywords:** browning, energy metabolism, 3,5-diiodo-l-thyronine, obesity, microRNA

## Abstract

The conversion of white adipose cells into beige adipose cells is known as browning, a process affecting energy metabolism. It has been shown that 3,5 diiodo-l-thyronine (T_2_), an endogenous metabolite of thyroid hormones, stimulates energy expenditure and a reduction in fat mass. In light of the above, the purpose of this study was to test whether in an animal model of fat accumulation, T_2_ has the potential to activate a browning process and to explore the underlying mechanism. Three groups of rats were used: (i) receiving a standard diet for 14 weeks; (ii) receiving a high-fat diet (HFD) for 14 weeks; and (iii) receiving a high fat diet for 10 weeks and being subsequently treated for four weeks with an HFD together with the administration of T_2_. We showed that T_2_ was able to induce a browning in the white adipose tissue of T_2_-treated rats. We also showed that some miRNA (miR133a and miR196a) and MAP kinase 6 were involved in this process. These results indicate that, among others, the browning may be another cellular/molecular mechanism by which T_2_ exerts its beneficial effects of contrast to overweight and of reduction of fat mass in rats subjected to HFD.

## 1. Introduction

Brown adipose tissue (BAT), prominently found in rodents and hibernating mammals, is specialized for the dissipation of chemical energy in the form of heat through the constitutively expressed uncoupling protein-1 (UCP1) [1]. BAT’s activation promotes an increase in the energy expenditure, counteracting obesity [2,3,4,5,6]. The discovery of this tissue in adult humans [7] and its association with multiple parameters of metabolic health [8,9,10,11,12] has led to interest in the potential to activate this tissue. The interest was further increased following the discovery that an intermediate category of adipocytes, known as beige or brite (brown in white), were identified in multiple white adipose tissue (WAT) fat pads in mammals [13,14,15]. Brite or beige adipocytes have a white fat-like phenotype, but acquire a brown fat-like phenotype when stimulated by physiological stimuli such as cold [16,17], exercise/dietary [18,19], certain hormones [17,20], or chemical treatments [21], possibly leading to increased thermogenesis [22,23]. This process is referred to as browning and was found to take place primarily in subcutaneous WAT (sWAT) [24,25,26]. The growing interest in brown and beige adipocytes comes from the ability of these cells to exert a significant impact on whole-body metabolism. Promoting beige/brite adipocyte biogenesis could be an ideal approach for treating metabolic abnormalities, such as obesity. Elucidating the physiology of beige/brite cells and identifying potential molecules or compounds that selectively act on their recruitment are interesting objectives to be achieved. Several studies have suggested that “browning” may be due to two main mechanisms: transdifferentiation of white adipocytes and de novo differentiation of beige/brite adipocytes [26]. Many genes and pathways involved in brown and beige adipocyte biology were suggested, including Prdm16 and certain miRNAs. Prdm16 is a critical regulator of brown adipocyte development and determines the thermogenic gene program in subcutaneous adipose tissue (sWAT). A recent study showed that the inhibition of miR-133a in BAT and sWAT upregulates Prdm16 and brown adipogenesis [27,28,29]. Moreover, miR-196a mediates the browning of white adipocytes by targeting Hoxc8, a repressor of the brown adipogenic marker C/EBPβ [29,30], and the downregulation of miR-34a induces browning gene expression (UCP1 and PRDM16) [31]. These studies indicate that microRNAs play important roles in brown adipose development and the browning of white adipocytes. As mentioned above, many factors, such as hormones, can induce the browning of WAT. Recently, several studies showed the involvement of the thyroid hormone (TH) in this intriguing process. It was shown that TH induces WAT browning through peripheral and central mechanisms [32,33,34,35,36,37], and evidence from animal and human studies revealed that TH disorders are associated with changes in both BAT thermogenesis and WAT browning (for review, see Weiner J et al., 2017) [37]. In addition, Matesanz et al. recently showed that MAPK kinase 6 (MKK6) controls T_3_-mediated browning in mice, identifying MKK6 as a central regulator of WAT browning [38].

In recent years, accumulating evidence has indicated that a thyroid hormone derivative, 3,5 diiodo-l-thyronine (T_2_), can increase the metabolic rate [39] and prevent high-fat diet-induced obesity [40,41]. Administration of T_2_ counteracted the occurrence of metabolic disorders associated with the prolonged intake of a high-fat diet (e.g., liver steatosis, hypertriglyceridemia, and insulin resistance) [40,41,42,43]. In a case study, T_2_ supplementation led to an increase of the resting metabolic rate and a body weight reduction in humans without undesirable side effects [44]. More recently, it was shown that T_2_ enhances glucose-induced insulin secretion in both rat β-cells and human islet cells [45].

The aim of this study was to investigate a possible effect of T_2_ on the browning of the subcutaneous WAT using high-fat diet-induced overweight rats and to attempt to elucidate the underlying mechanism of this effect. To rule out the effect of the housing temperature on white adipose browning, the rats were housed at thermoneutrality (28 °C), where sympathetic stimulation of facultative thermogenesis is depressed [33]. The importance of thermoneutral conditions was recently highlighted by Fisher and coworkers [46]. In fact, these researchers showed that, in metabolic studies, housing mice at thermoneutrality is necessary to align mouse energy metabolism to human energy metabolism and that housing temperature has a large impact on the outcome of such experiments and on their translatability to human situations. Considering the many metabolic activities shown by T_2_ and that at the lowest dose used, it is not associated with thyrotoxicity or undesirable cardiovascular side effects [47], new evidence of how this diiodothyronine affects energy expenditure may also be useful to stimulate an investigation on its potential use as an antilipidemic agent.

## 2. Materials and Methods

### 2.1. Animals and Animal Care

Animal care and experiments were conducted in accord with the guidelines of the Ethics Committee of the University of Campania “Luigi Vanvitelli”.

Every effort was made to minimize animal pain and suffering. Male Wistar rats (250–300 g, aged eight weeks) were kept one per cage in a temperature-controlled room at 28 °C (thermoneutrality for rats) under a 12-h light/12-h dark cycle. Before commencement of the study, a commercial mash (Charles River Laboratories, Calco, Italy) was available ad libitum and the animals had free access to water. At the start of the study (day 0), and after seven days of acclimatization to thermoneutrality, the rats were divided into three groups of five and treated as follows:

- The first group (N) received a standard diet (total metabolizable percentage of energy: 60.4 carbohydrates, 29 proteins, 10.6 fat J J^−1^; 15.88 kJ gross energy g^−1^; Muscedola, Milan, Italy) for fourteen weeks with a daily injection intraperitoneally of vehicle (saline), for the last four weeks;
- The second group (HFD) received a high-fat diet (280 g diet supplemented with 395 g of lyophilized lamb meat (Liomellin, Milan, Italy), 120 g cellulose (Sigma-Aldrich, St. Louis, MO, USA), 20 g mineral mix (ICN Biomedical, Solon, OH, USA), 7 g vitamin mix (ICN), and 200 g low-salt butter (Lurpak, Denmark); total metabolizable percentage of energy: 21 carbohydrates, 29 proteins, 50 fat J J^−1^; 19.85 kJ gross energy g^−1^) for fourteen weeks with a daily injection intraperitoneally of vehicle (saline), for the last four weeks;
- The third group (HFD-T_2_) received an HFD like the second group for 10 weeks and was subsequently treated for four weeks contemporary with HFD and T_2_ (50 μg/100 g BW).

All animals continued to have free access to water. At the end of the treatment, the rats were anesthetized using an intraperitoneal injection of chloral hydrate (40 mg 100 g^−1^ BW) and decapitated. Heart, gastrocnemius muscle, brown adipose tissue (BAT), and visceral white adipose tissues were excised and weighed. Subcutaneous white adipose tissue (sWAT) was excised and immediately frozen in liquid nitrogen and stored at −80 °C for later processing. The % of adiposity was measured as a ratio between visceral fat mass and body weight. Serum thyroid-stimulating hormone (TSH) levels were measured using a specific enzyme linked immunosorbent assay (ELISA) (TSH (Rodent) ELISA kit (Catalog No: KA2336, Abnova), according to the manufacturer’s protocol. Plasma concentrations of cholesterol were measured using a colorimetric enzymatic method employing a commercial kit (SGM Italia, Rome, Italy).

### 2.2. Histochemical Analysis

BAT and sWAT were dissected and fixed by immersing them in 4% formaldehyde in 0.1 M phosphate buffer overnight at 4 °C. Samples were dehydrated in ethanol, cleared, and embedded in paraffin blocks. Tissues were cut into serial 3-mm-thick sections and either stained with haematoxylin-eosin for morphological investigations or processed for immunohistochemical studies. For adipocyte area quantification, evaluations were performed on hematoxylin-eosin slides (sections every 400 µm) for each animal and at least 250 adipocytes per animal were analyzed. Sections were viewed with a Nikon Eclipse 80i light microscope (Nikon Instruments, Milan, Italy) at 20× magnification. Images were obtained with a Sony DS-5M camera connected to an ACT-2U image analyzer. The average of the adipocyte area and the frequency distribution were calculated from three sections per rat and at least three rats for each group (>750 adipocytes counted per group). Computed values were imported into Prism 5 (GraphPad Software, San Diego, CA, USA) for data analysis.

For immunohistochemistry, tissues were incubated with a primary polyclonal anti-rat UCP1 antibody raised in sheep (1:8000 dilution; generously provided by D. Ricquier, Paris, France). Immunoreactivity was assessed according to the avidin-biotin-peroxidase method with the aid of an ABC Vectastain-Elite Kit (Vector Labs, Burlingame, CA, USA), and peroxidase activity was revealed using 3,3′-diaminobenzidine tetrahydrochloride as the chromogen. Sections were counterstained with haematoxylin to reveal nuclei and mounted in Eukitt (Kindler, Freiburg, Germany). Omission of the primary antibody served as the negative control.

### 2.3. Insulin Tolerance Test

For the insulin tolerance test, rats fasted for 5 h and were injected intraperitoneally with insulin (homolog rapid-acting, 10 units/kg body wt in sterile saline; Novartis, Basel, Switzerland). Samples of blood were collected before the insulin tolerance test and at various times afterward (as indicated in the figures), and glucose values were determined by means of a glucose monitor (BRIO, Ascensia, NY, USA), calibrated for use with rats.

### 2.4. Preparation of Intrascapular BAT Lysate for Western Blotting

For western blot analysis, frozen intrascapular BAT was homogenized using an Ultra-turrax homogenizer in RIPA buffer (150 mMNaCl, 1.0% Triton X-100, 0.5% sodium deoxycholate, 0.1% SDS, 50 mMTris, pH 8.0) supplemented with 1 mM Na_3_VO_4_, 1 mM PMSF, and 1 mg/mL leupeptin. The BAT homogenate was left on ice for 1 h, during which time it was shaken every 10 min. The lysate was ultracentrifuged at 86,000× *g* for 10 min at 4 °C. To evaluate UCP1 levels, intrascapular BAT lysate (15 μg) was resuspended in SDS loading buffer and heated for 2 min at 90 °C. Protein from a single rat was loaded in each lane and electrophoresed on a 13% SDS-PAGE gel. UCP1 levels were detected in BAT lysates using anti-UCP1 (AB1426; Millipore-1:750 dilution). Stain-Free technology was used as normalization. TGX Stain-Free gels were activated for 1 min after SDS–electrophoresis and imaged using the ChemiDoc MP imaging system (Bio-Rad, Hercules, CA, USA) and ImageLab software (version 4.1, Bio-Rad). Stain-Free (after protein transfer) and fluorescent blot images were also collected using the ChemiDoc MP and ImageLab software. Analysis included the determination of total Stain-Free fluorescence and signal intensities of UCP1 of each sample lane on the blots with the “Lane and Bands” tool of ImageLab software. UCP1 signals were automatically normalized using ImageLab software with Stain-Free total lane volumes.

### 2.5. Preparation of Mitochondria

sWAT mitochondria were isolated after homogenization of the tissue in an isolation medium consisting of 220 mM mannitol, 70 mM sucrose, 20 mMTris–HCl, and 1 mM EDTA, pH 7.4 (all from Sigma-Aldrich Corp., St. Louis, MO, USA). The samples were centrifuged at 15,000× *g* for 30 min at 4 °C to get rid of the fat cushion. Moreover, the obtained pellet was resuspended in the same isolation medium and it was collected and transferred into new tubes for subsequent centrifugation at 500× *g* for 10 min at 4 °C. Subsequently, the supernatant was separated from the pellet and centrifuged at 15,000× *g* for 30 min at 4 °C. The obtained mitochondrial pellet was washed twice and was resuspended in 25 uL/20 uL of lysis buffer containing 20 mM Tris–HCl pH 7.5, 150 mM NaCl, 1 mM EDTA, 1 mM EGTA, 2.5 mM Na_2_H_2_P_2_O_7_, 1 mM b-CH_3_H_7_O_6_PNa_2_, 1 mM Na_3_VO_4_, 1 mM PMSF, 1 mg/mL leupeptin, and 1% Triton X-100 (all from Sigma-Aldrich and kept on ice). The protein content of each fraction was determined using Bio Rad’s DC method (Bio Rad Laboratories, Hercules, CA, USA).

### 2.6. Preparation of Total Lysates

For western blot analysis, sWAT was homogenized in the previously described lysis buffer using an Ultraturrax homogenizer and centrifuged at 16,000× *g* for 15 min at 4 °C. The protein concentrations of the supernatants of the centrifuged lysates were determined using Bio Rad’s DC method (Bio Rad Laboratories).

### 2.7. Western Blot Analysis

Mitochondrial lysate and total lysates were prepared by resuspending the mitochondria and the lysates in SDS loading buffer, as described by Laemmli [48], followed by heating for 5 min at 90 °C. Mitochondrial lysates containing 30 μg protein were loaded in each lane and were electrophoresed on a 12% SDS-PAGE gel. A polyclonal antibody against UCP1 (AB1426; Millipore-1:750 dilution) and an anti-rabbit antibody were used as primary and secondary antibodies, respectively, in a chemiluminescence protein-detection protocol (ECL, Advasta). Equal loading was verified using the Voltage-Dependent Anion channel (VDAC) as a control (PA1-954A, Thermo Scientific-1:1000). Total lysates containing 30 μg protein were loaded in each lane and were electrophoresed on a 10–12% SDS-PAGE gels. Polyclonal antibodies against PRDM16 (Abnova-1:1000 dilution), HOXC8 (Abcam-1:1000 dilution), MKK6 (Enzo Life Sciences-1:1000 dilution), p-38-Tot (Cell Signaling-1:1000 dilution), P-p-38 (Cell Signaling-1:1000 dilution), AMPK-Tot (Cell Signaling-1:1000), and P-AMPK (Cell Signaling-1:1000) and a monoclonal antibody against C/EBPβ (Abnova-1:1000 dilution), were used as primary antibodies. Equal loading was verified using β-Actin as a control (A2228, Sigma-Aldrich-1:1000).

### 2.8. Irisin Serum Determination

Irisin serum levels were determined using an Irisin Competitive ELISA kit (BioVision, San Francisco, USA) according to the manufacturer’s protocol.

### 2.9. miRNA and Total RNA Isolation

sWAT tissue samples were homogenized using a polytron in an appropriate volume of QIAzol lysis buffer (Qiagen, Hilden, Germany). miRNA and total RNA were extracted by the miRNeasy micro kit (Qiagen). MicroRNA-133a and microRNA196a were quantified along with RNU6B (reference transcript) by RT-qPCR with TaqMan^®^ miRNA assays from Applied Biosystems, according to the manufacturer’s protocol. Their expression levels were normalized to a reference gene (RNU6B) by using the 2^−ΔCt^ method. The analyses were performed on five independent experiments with each in triplicate.

The sWAT expression levels of the PGC1α and CIDEA were determined by RT-QPCR. In particular, 1 μg of total RNA was used to generate cDNA strands in a 20-μL-reaction volume using the Super Script First Strand Synthesis System for RT-PCR (Invitrogen). An equivalent of 25 ng total cDNA was subsequently used in the amplification. Real-Time quantitative RT-PCR (QRT-PCR) was carried out with 50 nM gene-specific primers and IQ SYBR Green supermix (Bio-Rad,) using standard cycle parameters on a MyiQ2 (BioRad). A melting curve analysis was completed following amplification from 55 to 95 °C to assure product identification and homogeneity. Each sample was repeated in triplicate and was normalized to the housekeeping gene β-actin to compensate for any differences in the starting quantity of total RNA.

PCR primers were designed by using the Primer 3 program (Untergasser et al., 2012), and synthesized and verified by sequencing at Eurofins Genomics (Ebersberg, Germany). Primers used were the following:

rβ-ACT sense 5′-GGA GAT TAC TGC CCT GGC TCC TA-3′
rβ-ACT Antisense 5′-GAC TCA TCG TAC TCC TGC TTG CTG-3′
rPGC1α sense 5′-AACCAGTACAACAATGAGCCCGC-3′
rPGC1α antisense 5′-TGAGGACCGCTAGCAAGTTTGC-3′
rCIDEA sense 5′-TTCCTCGGCTGTCTCAATGT-3′
rCIDEA antisense 5′- GCCCGCATAAACCAGGAAC-3′

### 2.10. Statistical Analysis

Data are expressed as the mean ± SEM and are normally distributed. The statistical significance of differences was determined using one-way analysis of variance (ANOVA) followed by the “Newman-Keuls Multiple Comparison Test”. Differences were considered statistically significant at *p* < 0.05.

## 3. Results

### 3.1. Administration of T_2_ to Rats Pre-Fed with a High-Fat Diet Modulates Adiposity without Inducing a Thyrotoxic State

The effect of T_2_ administration in rats pre-fed with a high-fat diet on body weight gain; on visceral adipose mass; on BAT, heart, and gastrocnemius weights; and on a TSH and cholesterol serum levels, are shown in Table 1.

**Figure 1:**
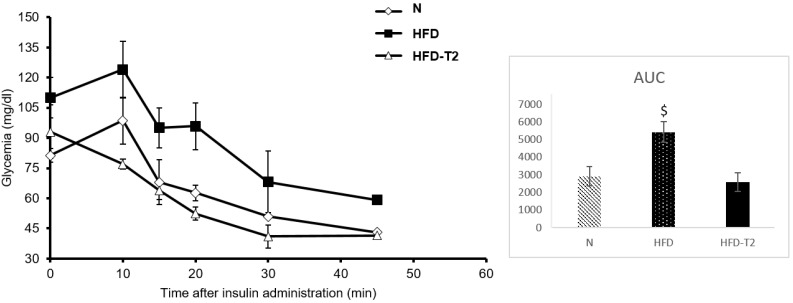
Insulin Tolerance test (ITT). Glycemia after insulin administration at different time points in N, HFD, and HFD + T_2_ rats. Area under the curve (AUC). Values are means ± SEM of five independent experiments (*N* = 5) $ *p* < 0.05 vs. N and HFD-T_2_. After 10 min from insulin injection, both N and HFD showed a small but not significant increase in glucose level, but this is not surprising and can happen frequently [49].

After 10 weeks of HFD, the contemporary T_2_ administration for the last four weeks of diet is effective in counteracting the further increase in adiposity. The HFD rats after 14 weeks of HFD gained about 60% more weight than N rats (+192.3 vs. 121 respectively). The HFD-T_2_ rats, conversely, showed less weight gain, with a value of 183.25 g (HFD-T_2_) vs. 192.3 g (HFD), corresponding to −5% (not significant). The visceral fat pad tissue weighed 30.61 per rat in HFD compared to 22.4 in HFD-T_2_ animals (*p* < 0.05; Table 1). The decreased amount of visceral fat pad observed in HFD-T_2_ rats corresponds to the reduced weight gain (−9 g and −8.2 g respectively). In HFD rats, we observed an increase of approximately 40% in BAT weight, and this weight is almost double that of N rats in HFD-T_2_ animals. These effects are not associated with a thyrotoxic state. The heart weights, TSH serum levels, free T_3_ serum levels, and free T_4_ serum levels did not significantly change from the normal euthyroid values, and the muscle mass increased in HFD rats and was not influenced by T_2_ administration (Table 1). Furthermore, the T_2_ could counteract the increase in cholesterol serum levels. In HFD-T_2_ rats, the serum cholesterol levels were reduced by ~23% compared to HFD rats (Table 1). The reduction in adiposity could be reflected in the improvement of glycaemic homeostasis. Insulin tolerance tests revealed that the reduction in glucose due to insulin administration was comparable for HFD-T_2_ and N animals, but was impaired in HFD animals (Figure 1).

These results reveal that the development of insulin resistance in overweight rats can completely revert after T_2_ administration. These effects were already shown by us in previous studies in similar models [41,50,51]. Here, we measured the same parameters in order to be sure that at the end of treatment with T_2_, the animals were in the same conditions as in previous studies. These conditions, in fact, are the optimal conditions to show the beneficial effects of T_2_ and to investigate the underlying mechanisms.

### 3.2. Effect of T_2_ Administration on BAT Morphology and on UCP1 Content

**Figure 2:**
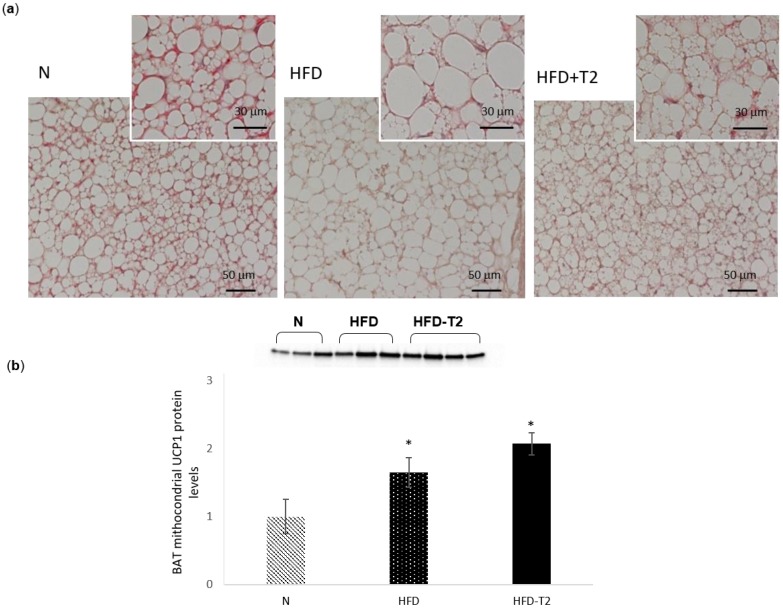
Effects of T_2_ administration on BAT morphology and on BAT UCP1 content. (**a**) Representative images (at low magnification-20×) showing different cellular organization within BAT parenchyma (haematoxylin and eosin staining). Insets: cellular shapes in the central part of the lobule in BAT parenchyma (40×), and (**b**) western blot analysis and quantification of the signals of UCP1 protein levels detected in the whole lysate. For total lysate, each lane contained 15 µg of protein from a single rat. Values are means ± SEM of five independent experiments (*N* = 5), * *p* < 0.05 vs. N.

Histological analysis of BAT showed that in HFD animals, the parenchyma displayed many large multilocular adipocytes that contained lipid droplets of an increased size compared to N droplets (Figure 2a). In T_2_-treated animals, multilocular brown adipocytes were uniformly distributed in parenchyma and appeared smaller in size than in HFD, showing an appearance comparable to that of controls, but with a more than doubled content of UCP1, as assessed by western blot analysis (Figure 2b).

### 3.3. T_2_ Administration Induces Browning of sWAT in Rats

**Figure 3:**
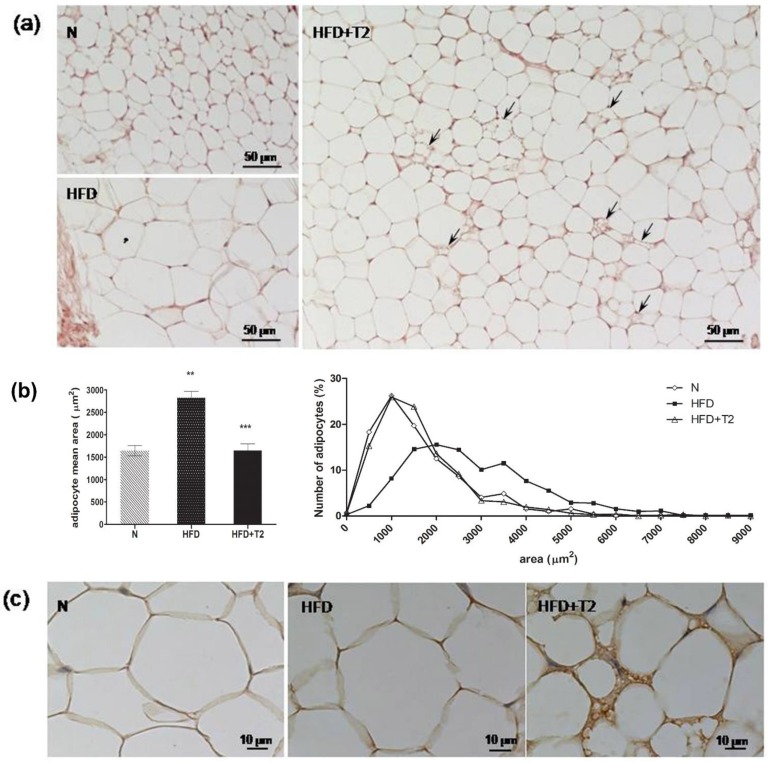
Effects of T_2_ administration on sWAT morphology. (**a**) Histological analysis, (**b**) morphometrical analysis, and (**c**) UCP1 immunohistochemistry of anterior sWAT of N, HFD, and HFD+T_2_ rats. Adipocytes from HFD rats are significantly larger than those from N rats. After T_2_ administration, the mean area and frequency distribution of the adipocytes were normalized to control values. A number of UCP1-stained paucilocular adipocytes are shown (arrows and panel (**c**)). More than 720 adipocytes were quantified from *N* = 3 rats from each group. Data are presented as Means ± SEM. ** *p* < 0.01 vs. N; *** *p* < 0.001 vs. HFD.

Subsequently, we performed a histological analysis of sWAT sections to verify whether T_2_ may stimulate browning. As shown in Figure 3a, adipocytes from HFD rats were uniformly larger than those from N rats. HFD + T_2_ sWAT showed large white adipocytes, but several adipocytes clearly appeared smaller than those of HFD rats. Morphometric analysis revealed that in rats treated with T_2_ contemporarily with the HFD for four weeks, following 10 weeks of HFD pre-feeding, the subcutaneous adipocytes mean area was significantly reduced with respect to that of rats continuously fed an HFD without T_2_ treatment and reverted to control (N) value (Figure 3b). The mean area was 1651 ± 146.8 µm^2^ (HFD + T_2_) vs. 2827 ± 145.5 µm^2^ (HFD) and 1646 ± 115.2 µm^2^ (N). Animals from the HFD group presented a higher percentage (44%) of large adipocytes (≥2000 µm^2^) when compared to the control group (13%). T_2_ treatment normalized the adipocytes profile because it significantly increased the percentage of small adipocytes. HFD+T_2_ increased the percentage of adipocytes below 2000 µm^2^ (89%), similar to that from the N group (87%), and reduced the large adipocytes (11%). Surprisingly, a number of paucilocular and multilocular adipocytes appeared intermixed into the white parenchyma, and most of them were UCP1 immunoreactive (Figure 3c).

**Figure 4:**
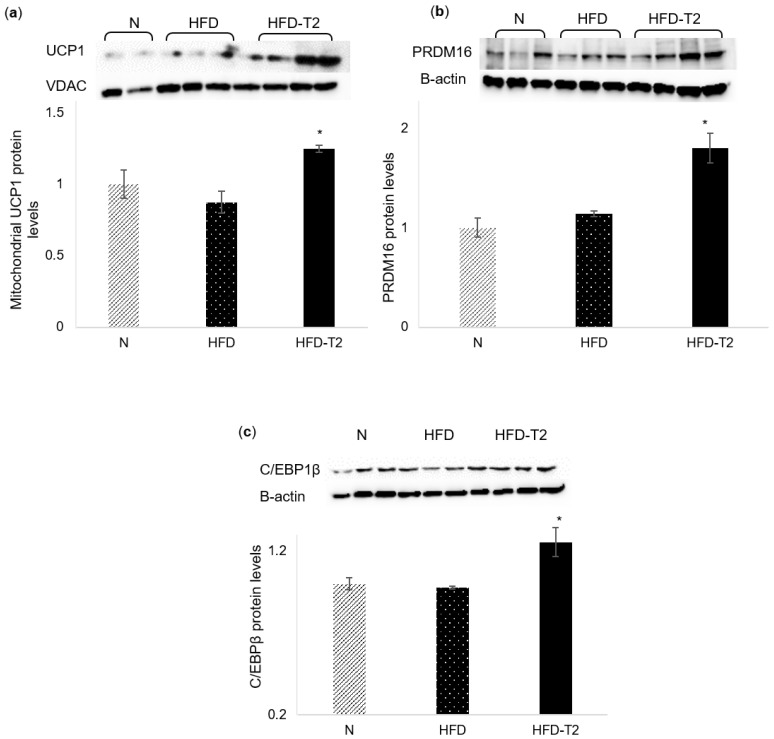
Effects of T_2_ administration on expression of the main browning genes in sWAT. Western blot images and densitometry showing relative expression of the main browning genes in sWAT of rats. (**a**) UCP1, Voltage-dependent Anion Channel (VDAC) in mitochondrial of sWAT, and (**b**) PRDM16, (**c**) and C/EBPβ and β-actin in sWAT lysates. Values are means ± SEM of five independent experiments (*N* = 5), * *p* < 0.05 vs. N-HFD.

Based on this evidence, we performed a screening of the main browning markers and measured their protein levels. Our results showed that the expressions of uncoupling protein 1 (UCP1), PR domain containing 16 (PRDM16), and CCAAT/enhancer-binding protein beta (C/EBPβ) were significantly enhanced in the sWAT of HFD-T_2_ rats compared to HFD rats (Figure 4). In Figure 4a, it is possible to observe the weak signal of UCP1 in the sWAT of both N and HFD animals, whereas in T_2_-treated rats, the signal becomes much more pronounced. PRDM16 is involved in both the induction of BAT genes and the repression of WAT genes. Indeed, PRDM16 can interact with the promoter regions of various WAT genes to repress their expression. Conversely, PRDM16 induces BAT-specific genes by associating with the transcriptional coactivators PGC-1α and PGC-1β [52].

**Figure 5:**
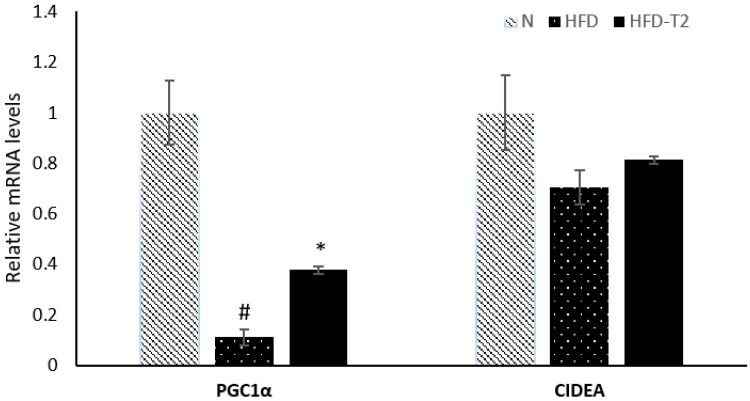
qPCR analysis of PGC1α and CIDEA in sWAT of N, HFD, and HFD-T_2_ rats. Values are means ± SEM of five independent experiments (*N* = 5), ^#^ *p* < 0.05 vs. N; * *p* < 0.05 vs. N-HFD.

In fact, the PGC1α expression levels are significantly enhanced in HFD-T_2_ rats when compared to HFD animals (Figure 5). The expression levels of cell death-inducing DNA fragmentation factor alpha-like effector A (CIDEA), another important browning regulator, are slightly not significantly increased by about 13% in rats treated with T_2_ when compared to rats fed a high fat diet (Figure 5).

### 3.4. T_2_ Administration Induces Downregulation of miR-133a, Resulting in Up-Regulation of PRDM16

**Figure 6:**
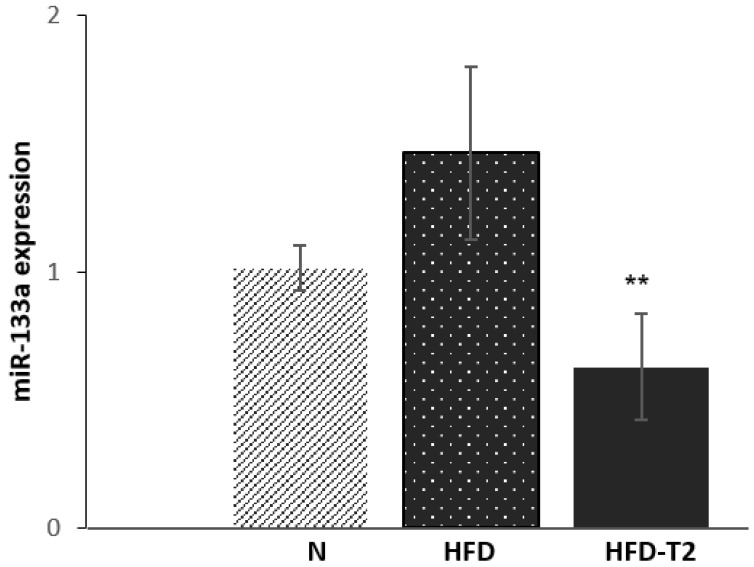
qPCR analysis of miR-133a in sWAT of N, HFD, and HFD-T_2_ rats. Values are means ± SEM of five independent experiments (*N* = 5), ** *p* < 0.05 vs. N-HFD.

Subsequently, we investigated the possible pathways involved in this process. As shown before, T_2_ can stimulate the protein levels of the main regulator of brown adipocyte development, including PRDM16. The 3′ UTR of PRDM16 mRNA is targeted by a microRNA, known as miR-133a, that is known to negatively regulate its transcript [53,54]. Indeed, miR-133a is one of the many identified miRNAs involved in the browning process [29]. Our next aim was to evaluate the miR-133a’s possible involvement in T_2_ induction of browning. Consistent with our hypothesis, we found that T_2_ administration to HFD-fed rats significantly reduced miR-133a expression levels compared to HFD-fed rats alone (Figure 6).

Taken together, these results suggest that T_2_ activates browning in sWAT, at least partially, via regulating miR-133a expression levels.

### 3.5. T_2_ Administration Induces Up-Regulation of miR-196a, Resulting in Down-Regulation of HOXC8

**Figure 7:**
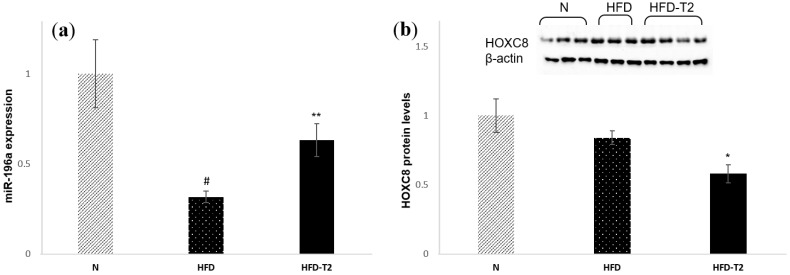
qPCR analysis of miR-196a in sWAT of N, HFD, and HFD-T_2_ rats. (**a**) qPCR analysis of miR-196a in sWAT of N, HFD, and HFD-T_2_ rats; (**b**) Immunoblots and densitometry showing relative expression of HOXC8 and β-actin in sWAT lysates. Values are means ± SEM of five independent experiments (*N* = 5), * *p* < 0.05 vs. N-HFD; ** *p* < 0.05 vs. HFD ^#^ *p* < 0.05 vs. N.

We previously showed that T_2_ administration results in upregulation of the protein levels of c/EBPβ, one of the master regulators of brown adipogenesis. HOXC8, which is highly expressed in WAT, represses the c/EBPβ mRNA. Moreover, HOXC8 is negatively regulated by miR-196a [29]. Interestingly, we found that the administration of T_2_ to HFD-fed rats could modulate the expression of miR-196a. Indeed, our results showed that Hoxc8 protein levels are significantly reduced in HFD + T_2_ rats compared to HFD alone (Figure 7b). Accordingly, the expression levels of miR-196a were increased in HFD + T_2_ rats compared to HFD (Figure 7a).

### 3.6. T_2_ Administration Reduces the Phosphorylation of an Important Regulator of Browning: MAPK Kinase 6 (MKK6)

**Figure 8:**
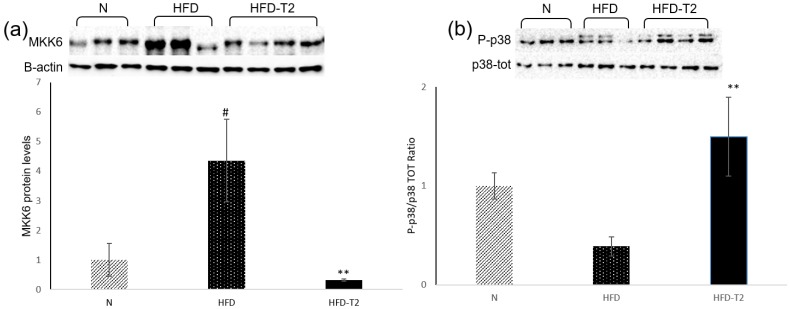
Effects of T_2_ administration on MKK6 and P-p38 in sWAT. (**a**,**b**) Immunoblot and densitometry showing relative expression of MKK6, P-p38, and total p-38 in sWAT lysates. Values are means ± SEM of five independent experiments. *N* = 5, ^#^ *p* < 0.05 vs. N; ** *p* < 0.05 vs. HFD.

Then, we investigated the role of the upstream p38 activator MAPK kinase 6 (MKK6) in obesity induced by a high-fat diet (HFD). MKK6 seems to be an important regulator of browning. The lack of MKK6 increases the basal expression of UCP1 and promotes T_3_-mediated induction of UCP1 expression in WAT. Moreover, the browning of WAT and subsequent increased energy expenditure in mice lacking MKK6 protects these animals against HFD-induced obesity [38]. From our analysis, the MKK6 protein levels were upregulated in HFD-fed rats and downregulated in HFD + T_2_ rats (Figure 8a).

We also measured the phosphorylation of MAP kinase p38, since cell culture studies with p38 inhibitors identified this kinase as a possible mediator of UCP1 expression in the browning [55,56]. Interestingly, we found that the phosphorylation of p38 MAPK tended to be decreased in HFD compared to N rats and upregulated by T_2_ administration to HFD rats (Figure 8b).

### 3.7. T_2_ Administration Increased the Serum Levels of Irisin

**Figure 9:**
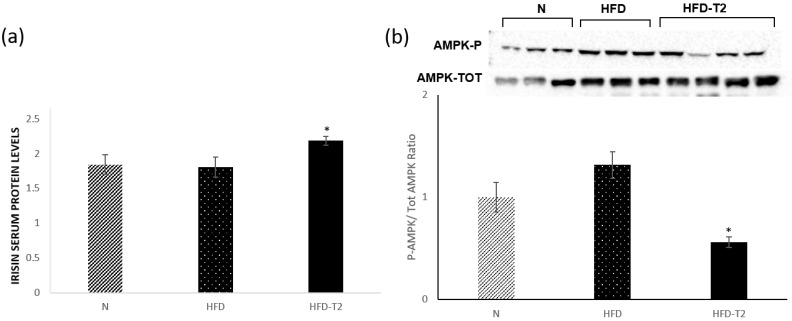
Effects of T_2_ administration on Irisin and P-AMPK in sWAT. (**a**) Irisin serum levels detected by a competitive Enzyme Linked-Immunosorbent Assay (ELISA) and (**b**) immunoblot and densitometry showing relative expression of P-AMPK and total AMPK in sWAT lysates. Values are means ± SEM of five independent experiments. *N* = 5, * *p* < 0.05 vs. N and HFD.

Finally, to verify if p38 can be triggered by an alternative pathway, involving, for example, the AMPK and TAB1/TAK1 complex [38,57] or Irisin [56], we measured the phosphorylation levels of AMPK and the serum levels of Irisin. Our data showed that Irisin serum levels were significantly increased in HFD+T_2_ rats (of about 30%) when compared to rats fed HFD (Figure 9a), while the phosphorylation levels of AMPK were reduced in the sWAT of HFD-T_2_ rats when compared to both N and HFD animals (Figure 9b).

## 4. Discussion

In the last several years, many studies have focused on thyroid hormone mimetics that selectively increase energy expenditure [58,59]. Particular attention was focused on 3,5 diiodo-l-thyronine as a “fat burning” agent because of its ability to increase both fatty acid oxidation and the metabolic rate by primarily non-genomic activity. Indeed, T_2_ has a weak transactivating capacity of TRβ target genes both in vitro and in vivo [41,60,61,62]. Most studies on T_2_ metabolic effects have focused on liver and skeletal muscle and shown that T_2_ can prevent body weight gain when administered simultaneously to rats fed a high-fat diet without inducing T_3_-related undesirable side effects (tachycardia, cardiac hyperplasia, muscle wasting) [40,47,51]. By stimulating the hepatic FA oxidation rate, T_2_ efficiently prevented HFD-induced hepatic fat accumulation and an increase in serum triglycerides (TGs) and cholesterol levels [40,41,50,51]. An important consequence of these effects included increased skeletal muscle insulin sensitivity due to an increased response to insulin and structural shifts toward glycolytic myofibers [51]. In this study, we show that T_2_ induces the browning of sWAT in rats housed at thermoneutrality. Although the sWAT tissue from the inguinal area is the classical fat pad where a large part of browning studies were performed, it was also described in other depots, including the gonadal one [63,64,65,66,67,68,69]. It was recently demonstrated that when browning was centrally induced, gWAT and inguinal sWAT were affected to a similar extent [66,68]. In the present study, T_2_ was administered for 30 days in high-fat-diet-induced overweight rats (after 10 weeks of HFD), and the metabolic results are in line with those of our previous studies obtained through a shorter treatment and/or with a concomitant administration of T_2_ [40,41,50,51]. Indeed, our data now show that T_2_, when administered to rats treated with HFD for 10 weeks and then for another four weeks with HFD plus T_2_, has the potential to counteract an increase of adiposity without inducing thyrotoxicosis. The HFD-T_2_ rats accumulated much less fat in their adipose tissue than the HFD animals. In addition, these rats showed reduced increases in cholesterol serum levels and the absence of the insulin-resistance induced by HFD. The heart weight and TSH serum levels were nearly the same in all groups. While confirming the beneficial effects of T_2_ in rats, in partial contrast with our data, Padron et al. showed that the daily administration of T_2_, at the same dose, induced a significant decrease of about 20% in TSH levels and a state of hypothyroidism [42]. A possible explanation for this apparent discrepancy is that (a) the duration of the treatment was 90 days instead 30 days; (b) they used chow fed rats instead of HFD fed animals; and most importantly, (c) the housed temperature was 23 °C, while in our case, the animals were housed at thermoneutrality (28 °C). This last difference is important in metabolic studies. Rats housed at 23 °C are mildly cold-exposed, with the consequent activation of the adrenergic system and therefore of the subsequent influences on the metabolism, which can alter the results caused by the administration of T_2_.

The ability of T_2_ to affect thermogenesis may also be associated with changes in adipocyte morphology and functionality. In the BAT, we showed that T_2_ administration restored a morphology similar to the N ones, but with more than double the content of UCP1, inducing BAT to assume a classical role consisting of counteracting the development of diet-inducing overweight. Our histological data show that T_2_ promotes the browning of WAT. This browning was observed in a section of the anterior sWAT of HFD-T_2_ rats in which several white adipocytes change their phenotype. These adipocytes acquire a multilocular phenotype instead of their classic unilocular morphology, also showing UCP1 immunoreactivity. To verify the effect of T_2_ on browning, we measured the expression levels of some browning markers, such as UCP1, PRDM16, C/EBPβ, PCC1α, and CIDEA in sWAT. The results have shown that the expression of these markers is significantly increased in rats treated with T_2_, except for CIDEA, whose expression levels are only slightly and not significantly increased in HFD-T_2_ rats (+13%). We investigated other possible pathways involved in this intriguing process. As mentioned, recent studies identified several miRNAs involved in browning. Interestingly, we identified two miRNAs as T_2_-targets. One is miR-133a, which targets the 3′ UTR of Prdm16 transcripts, repressing its expression. Consistent with this result, miR-133a was significantly downregulated in the sWAT of HFD-T_2_ rats compared to HFD rats, showing that T_2_ could regulate PRDM16 expression through modulation of the expression of this miRNA.

The other miRNA is miR-196a. This miRNA’s target gene is white-fat gene Hoxc8, homeobox c8, highly expressed in WAT and categorized as a white-fat gene. Adipose-specific expression of miR-196a results in the enhanced browning of WAT and protects mice from HFD-induced obesity and insulin resistance. It was found that Hoxc8 represses c/EBPβ expression. This gene’s expression is significantly increased in the sWAT of HFD-T_2_ rats compared to HFD rats, which is associated with reduction in protein levels of Hoxc8 and with increases in the protein levels of C/EBPβ. Finally, we investigated the role of MAPK Kinase 6 (MKK6), considered a central regulator of WAT browning and a possible target for obesity treatment. Indeed, mice lacking MKK6 show the browning of WAT and subsequent increased energy expenditure, and are protected against HFD-induced obesity. This phenotype depends on T_3_ signaling: the lack of MKK6 increases the sensitivity of adipose tissue to T_3_-mediated browning [38]. Our results showed that in HFD-T_2_ rats, the MKK6 phosphorylation levels are significantly downregulated in accordance with the browning induction by T_2_. These data seem to disagree with the increased phosphorylation levels of p-38, of which MKK6 is a canonical activator [70]. It was postulated that in the absence of canonical activation, p38 can be triggered by an alternative pathway involving, for example, the AMPK and TAB1/TAK1 complex [38,57] or Irisin [56]. In this study, we measured the phosphorylation levels of AMPK (P-AMPK) and serum levels of Irisin. While the levels of P-AMPK were unaffected by T_2_ administration, a significant increase in irisin serum levels was observed. These data suggest that the Irisin could be involved in this process. However, to accept this possibility, further, detailed studies are needed.

In conclusion, these data demonstrate that the effect of the T_2_ on BAT metabolic features may also be associated with changes in adipocyte morphology. In inguinal sWAT, the T_2_ acts primarily in adipogenesis and browning. The browning of WAT involves different pathways. To the best of our knowledge, this study is the first that demonstrates the efficacy of T_2_ in the induction of sWAT browning. In addition, considering all its beneficial effects and that much research is still needed, these new findings could stimulate studies aimed at demonstrating the possible use of T_2_ as a therapeutic agent.

## Figures and Tables

**Table 1:** Body weight gain together with WAT, BAT, heart, muscle weights and serum TSH, FT3, FT4 and cholesterol levels in N, HFD and HFD-T_2_ rats.

Parameters	N (14 Weeks)	HFD (14 Weeks)	HFD-T_2_ (10 Weeks + 4 Weeks)
**BW gain (g)**	121 ± 13.65	192.3 ± 8.08 *	183.25 ± 9.4 *
**WW (g)**	10.85 ± 0.98	30.67 ± 1.80 *	22.4 ± 1.76 **
**% adip.**	3.19 ± 0.26	7.24 ± 0.45 *	5.24 ± 0.32 **
**BT (g)**	0.49 ± 0.068	0.70 ± 0.028	0.83 ± 0.080 *
**HW (g)**	1.08 ± 0.097	1.14 ± 0.104	1.18 ± 0.039
**GW (g)**	1.83 ± 0.045	2.36 ± 0.088 *	2.37 ± 0.064 *
**TSH (μU/mL)**	0.0065 ± 0.00028	0.007 ± 0.0011	0.0068 ± 0.00048
**Free T_3_ (pg/mL)**	1.86 ± 0.2	1.94 ± 0.3	1.81 ± 0.2
**Free T4 (ng/mL)**	0.91 ± 0.08	0.88 ± 0.07	0.79 ± 0.09
**Cholesterol (mg/dL)**	40.66 ± 2.72	74.66 ± 2.84 *	57.33 ± 4.63 **

Body Weight (BW) gain, WAT weight (WW), %adiposity, BAT weight (BT), Heart Weight (HW), gastrocnemius weight (GW), thyroid-stimulating hormone (TSH), free T_3_, free T4, and cholesterol serum levels in rats fed a standard diet (N), high-fat diet (HFD), and HFD with contemporary administration of T_2_ (HFD-T_2_). * *p* < 0.05 vs. N; ** *p* < 0.05 vs. N and HFD. Values are mean ± SEM of five independent treatments.
